# Acknowledgement to Reviewers of *Healthcare* in 2018

**DOI:** 10.3390/healthcare7010007

**Published:** 2019-01-09

**Authors:** Healthcare Editorial Office

**Affiliations:** MDPI, St. Alban-Anlage 66, 4052 Basel, Switzerland

Rigorous peer-review is the corner-stone of high-quality academic publishing. The editorial team greatly appreciates the reviewers who contributed their knowledge and expertise to the journal’s editorial process over the past 12 months. In 2018, a total of 146 papers were published in the journal, with a median time to first decision of 20 days and a median time to publication of 43.5 days. The editors would like to express their sincere gratitude to the following reviewers for their cooperation and dedication in 2018:
Abdi, HamidrezaLissenden, BrettAdam, SalomeLiu, KarenAgrawal, PriyankaLloyd, VettAhern, HollyLochhead, RobertAitken, DominicLopez, NanetteAlberdi, PilarLópez-López, DanielAllen, VivietteLorenzo Bermejo, JustoAlsharairi, NaserLucero, JessAmagai, TeruyoshiLundman, BeritAndrews, AnyaLutfiyya, NawalAndy, Uduak UmohMacias-Nuñez, JuanAnsa, BenjaminMacLeod, RoderickArcury, Thomas A.Madsen, MarieAshish, PathakMagnavita, NicolaAssari, ShervinMakate, MarshallBai, RenrenMalave-Llamas, KarloBailey, KylieMarsh, PaulineBarone, Daniel A.Martelletti, PaoloBarrie, HelenMartin, ColleenBarzilay, Joshua IMartino Alba, RicardoBay, Jacquie L.Mason, LauraBazina, MohamedMathew, MonaBecker, KathrinMathews, Anne E.Beck-Sague, Consuelo M.Mathur, ManuBekmuratova, SarbinazMcCoy, PamelaBell, Carl C.McCullough, JulieBennett, William E.McDarby, VincentBerkowsky, RonaldMcmorrow, TaraBernardi, SaraMedina, MiguelBianchi, VittorioMickevičienė, AušraBingham, RichardMiddelveen, MarianneBjørge, HeidiMiddlecamp, CathyBlack, Helen K.Mignon, SylviaBlumrosen, GaddiMilte, RachelBodenheimer, ThomasMinicozzi, PamelaBoelsbjerg, Hanne BessMistri, AmitBonham, JimMitchell, JohnBoomer, Jonathan S.Mix, KimberleeBotturi, AndreaModell, Stephen M.Bouwmans, JogéMoore, PhilipBranney, JonnyMorgan, MikeBransfield, RobertMorgante, GiuseppeBreslau, JoshuaMorita, MitsuhiroBreuninger, MatthewMorris, Meg E.Brubacher, Laura JaneMulcahy, RoryBrzoska, PatrickMurphy, Dominic M.Bulkeley, KimMurray-Leisure, KatherineBurrascano, Joseph J.Muscat, DanielleBusuttil, WalterNakanishi, MiharuCaldwell, CurtisNaoki, MiyazatoCallister, RobinNarasimhan, SukanyaCameron, DanielNarasimhulu, Chandrakala AlugantiCangkrama, MichaelNatale, VincenzoCaspi, YaelNikles, Catherine J.Catherwood, Philip A.Nwanodi, OromaCeccato, AdriánOlivas, Raul NavarroChakraborty, RajaOmar, Syed HarisChang, Chin-ShunOneto, LucaChapple, HelenOrtega Avila, Ana BelenChatwood, SusanPace, NelsonChau, Hing-WahPagano, IanChau, Kwok-wingPal, TuyaChaudhury, HabibPalioura, SotiriaCheemarla, NagarjunaPan, XiChen, YanPapazafiropoulou, AthanasiaCheng, Tsang-HsiangPappas, Christopher J.Ciaccio, MarcelloParr, EvelynClaros, EdithParthasarathy, RaghuveerClavenna, AntonioPatel, MeghnaClay, Olivio J.Patridge, StephanieCleary, Margot P.Patterson, KaitlinClement, FionaPatterson, Susan LibrizziColeman, Karen J.Paul, BimolComas, MariaPentaris, PanagiotisCorkery, Marie B.Pérez-Fuentes, María Del CarmenCrawford, DerekPeri-Rotem, NitzanCuberos, Ramón ChacónPleasants, Roy A.Cutler, Sally JanePotvin, NoahCuviello, AndreaPrunuske, AmyDe Gisi, SabinoPuri, BasantDeBeer, Bryann B.Raffenaud, AmandaDeFelice, Nicholas B.Rahbel RahmanDeligeoroglou, EfthimiosRahman, MilladurDibaba, DanielRainisch, BethanyDisler, RebeccaRamírez-Vélez, RobinsonDoney, AlexanderRamtekkar, UjjwalDotov, DobromirRaposo, MariaDownes, Loureen SmartRasiah, RohanDowning, JuliaReeve, KathleenDu Preez, JaniceReif, Kathryn E.Dubey, HarishchandraRen, QingzhongDuquette, DebraRezaeihemami, MohsenEl-Safty, Sherif A.Rigby, Brandon RhettEvans, MaryRoy, RajshriEwen, Heidi HRuiz-Fernández, María DoloresFacal, DavidSahay, BikashFajardo-Bullón, FernandoSalahuddin, MelihaFalk, Nancy L.Sallis, AmandaFallon, BrianSandhi, ArifinFarney, Tyler MSan-Segundo, RubénFingerhut, RalphSantulli, GaetanoFiocco, AlexandraSapezinskiene, LaimaFirmino, HoracioSapi, EvaFisher, KarenSato, EiichiFlott, Elizabeth A.Sayer, R. DrewFonteh, AlfredScarlat, Marius M.Francis, JoelSchaefer, MichaelFrank, KristynSchlieber, MarisaFrontoni, EmanueleSchmid, ChristopherGadd, HollySchotthoefer, Anna M.Gao, Shiqian SherryScialla, Timothy JudeGaudiano, BrandonSearle, Samuel D.Geldmacher, DavidSelvamani, AmuthaGhosh, SantoshSenbonmatsu, TakaakiGianni, PesSerafini, GianlucaGill, Pritmohinder S.Setty, KarenGill, TiffanyShah, JyotsnaGirardis, MassimoShah, Rachana D.Gitau, MaryShapiro, Eugene D.Götz, TheresaShatte, AdrianGou, ZhonghuaShenoi, Asha N.Graham, RyanShityakov, SergeyGraven, VibekeShoji, KotaroGravina, RaffaeleShort, LeonieGreenberg, RosalieSibinga, EricaGriffith, DerekSiganos, Charalambos S.Gulino, MatteoSilva, AbigailGupta, OlgaSingh, VirtajGustafsson, SusanneSlusky, DavidGuthrie, DawnSmall, RoyGuthrie, RobertSmith, Andrew J.Gyawali, PradipSmith, BrandtHain, RichardSmith, Heather F.Halek, MargaretaSmith, Jared GHalperin, JohnSmith, JessicaHan, JayoungSoloski, Mark J.Hanley, JanetSperber, Nina R.Harding, MaireadSpratt, JenniferHaring, RobinSpreat, ScottHarley, DavidStegemoller, ElizabethHartung, John G.Stiller, Carl-OlavHattori, ToshioStocks, CarolHeath, GemmaStowers, Kristen CookseyHeima, MasahiroStrobino, BarbaraHemming, RebeccaSwamy, GaneshHerbert, AnthonySwango-Wilson, AmyHicks, James KevinSweeney, TimothyHirsch, Annemarie G.Szewczuk, Myron R.Ho, EnochTaff, Derrick B.Hodges, LynetteTalati, ArdesheerHolefors, AnnaThapa, SantoshHong, YonggeunThompson, Lisa MarieHorowitz, RichardTiwari, PoojaHosohata, KeikoToledo, AlvaroHouse, Darlene R.Tolhurst, EdwardHsin, Jennifer I-ChinTonacci, AlessandroHunfeld, Klaus-PeterTorres Vaamonde, Jose EnriqueHwang, AmieTovey, RobIzawa, Kazuhiro P.Traini, ToninoJacob, JennaTrepanier, AngelaJamie, KimberlyTurner, AndyJanakiraman, HarinarayananTykkyläinen, MarkkuJayaram, LataVaccaro, Joan A.Jimenez, Daniel EnriqueVaismoradi, MojtabaJohnson, SamVan Hoof, JoostJozwiak, MathieuVardasca, RicardoKachadourian, Lorig KVashist, Sandeep K.Karim, Mohammad RezaulVears, DanyaKarim, NasiaraVelosa, TeresaKennel, JulieVenderbos, LionneKent, BrianneVidic, JasminaKevern, PeterVignon, PhilippeKeynan, IritVolpe, StellaKidd, Lisa A.Vozza, IoleKim, WangdoWang, Chun-HouKjemtrup, AnneWark, StuartKobayashi, AkiraWarlow, Timothy A.Kocot, JoannaWashington, David MichaelKonradsen, HanneWeber, Ellerie SKowal, MałgorzataWeiner, Shira SchecterKrick, StefanieWhite, FranklinKristensen, TroelsWilliams, CarolynKruse, Robin L.Williams, MyiaKucharska-Newton, AnnaWilson, CristinaKumar, DevenderWilson, PeterKumar, GauravWinbolt, MargaretKvaal, KariWong, Judith Ju-MingKypriotakis, GeorgeWright, BradLachenmeier, DirkYamamoto, MiwaLane, AtheneYao, YuChunLange, EwaYatera, KazuhiroLara-Otero, KarlenaYau, YungLawson, WilliamYoo, Terry S.Lee, Paul H.Young, JeanineLee, SangwooYu, Tsung-HsienLeitão, Jorge H.Yuan, XueLewis, Jordan P.Zavattaro, ElisaLiegner, KennethZhao, LinpingLin, Pao-HwaZhou, XujuanLincoln, KarenZurita Ortega, FélixLindor, Noralane M.



